# Zonal variation in primary cilia elongation correlates with localized biomechanical degradation in stress deprived tendon

**DOI:** 10.1002/jor.23229

**Published:** 2016-03-23

**Authors:** Daniel Rowson, Martin M. Knight, Hazel R.C. Screen

**Affiliations:** ^1^ Institute of Bioengineering and School of Engineering and Materials ScienceQueen Mary University of LondonMile End RoadLondonE1 4NSUnited Kingdom

**Keywords:** mechanobiology, inter‐fascicular matrix, tendinopathy, mechanotransduction

## Abstract

Tenocytes express primary cilia, which elongate when tendon is maintained in the absence of biomechanical load. Previous work indicates differences in the morphology and metabolism of the tenocytes in the tendon fascicular matrix (FM) and the inter‐fascicular matrix (IFM). This study tests the hypothesis that primary cilia in these two regions respond differently to stress deprivation and that this is associated with differences in the biomechanical degradation of the extracellular matrix. Rat tail tendon fascicles were examined over a 7‐day period of either stress deprivation or static load. Seven days of stress deprivation induced cilia elongation in both regions. However, elongation was greater in the IFM compared to the FM. Stress deprivation also induced a loss of biomechanical integrity, primarily in the IFM. Static loading reduced both the biomechanical degradation and cilia elongation. The different responses to stress deprivation in the two tendon regions are likely to be important for the aetiology of tendinopathy. Furthermore, these data suggest that primary cilia elongate in response to biomechanical degradation rather than simply the removal of load. This response to degradation is likely to have important consequences for cilia signalling in tendon and as well as in other connective tissues. © 2016 The Authors. *Journal of Orthopaedic Research* Published by Wiley Periodicals, Inc. on behalf of Orthopaedic Research Society. J Orthop Res 34:2146–2153, 2016.

Tendons perform the primary function of transferring force from muscle to bone, undergoing regular cyclic loading, and constant pre‐stress as a result of muscle attachment. Tendons are easily injured and chronic tendon disease, known as tendinopathy, is both prevalent and poorly understood. Tendon has long been known to remodel its extracellular matrix (ECM) in response to changing loading conditions; behavior critical for tendon homeostasis and to retain normal function.^1^ However, while external stimuli are essential for healthy tendon maintenance, alterations in loading are also thought to be a significant factor in the development of tendinopathy. Overload has been shown to generate a catabolic cellular response in tendon,^2^ while the complete removal of external stress, known as stress deprivation or shielding, also results in degeneration of the tendon, including changes in collagen organization and mechanical properties similar to the effects of tendinopathy.^3^, ^4^, ^5^ Stress deprivation in tendon can occur due to a number of different pathologies, such as tumors, infection, congenital deformities, degenerative diseases, and trauma.^6^ It has also been hypothesized that the fibrillar micro damage resulting from overuse of tendon results in local areas of stress deprivation, and it is the subsequent stress deprivation conditions that are responsible for the development of tendinopathy.^7^

Tendons are hierarchical structures, composed at the macroscale of collagen rich fascicles, bound together by a softer highly hydrated matrix called the endotenon or inter‐fascicular matrix (IFM). Both the collagen rich fascicular matrix and the IFM contain tenocytes which synthesize and maintain the extracellular matrix. However, there are morphological and metabolic differences between the populations of cells in the IFM and FM, with rounder, more abundant and more active cells in the IFM.^2^ In addition, work from our group has shown that cyclic overloading of tendon tissue results in increased expression of inflammatory markers and catabolic proteins such as MMPs in the cells of the IFM, when compared with those in the fascicular matrix.^8^ However, differences between these regions have not been investigated under stress deprivation. Figure 1 shows a tendon fascicle in cross section.

**Figure 1:**
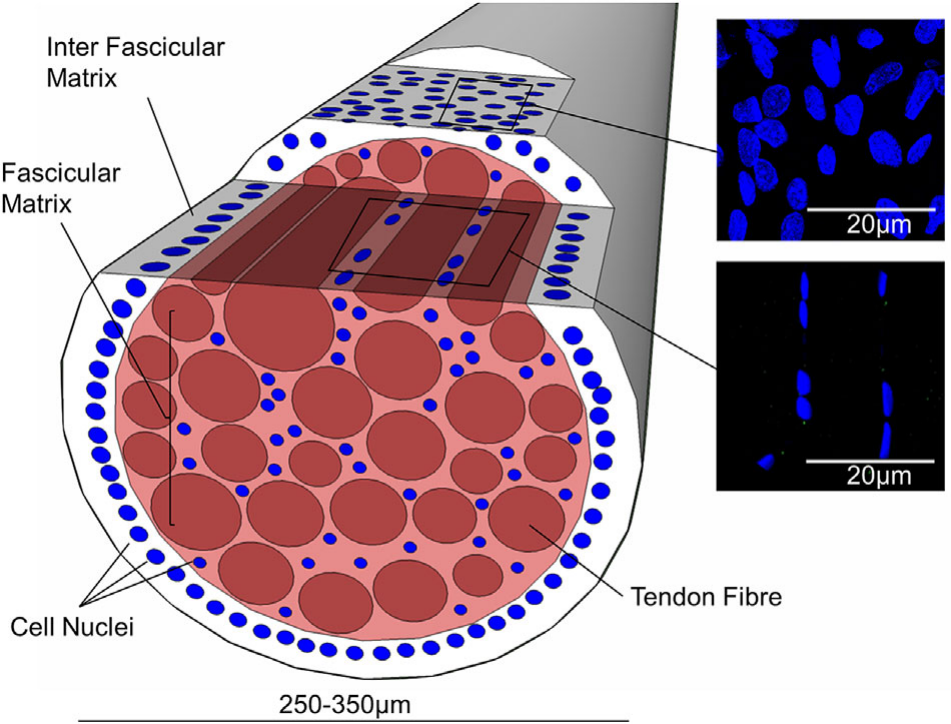
Schematic showing a fascicle; highlighting the imaging planes in which confocal images are taken of the fascicular and inter‐fascicular matrix. The images on the right show representative images of nuclei and cilia in the inter‐fascicular and fascicular matrix.

Primary cilia are thin, non‐motile, eukaryotic cellular organelles, which project out from the cell, with almost every cell in the human body containing a single cilium.^9^ Primary cilia are composed of an array of nine microtubule doublets enclosed by a specialized cell membrane.^9^, ^10^ The length of cilia and the proteomal content are tightly controlled by interflagellar transport and cilia are usually between 1 and 2 μm in length in fresh tendon. First observed by Kowalevsky^11^ in 1867 primary cilia were mostly ignored for the next 100 years.^12^ However, over the last 20–30 years a growing interest in their behavior has led to the discovery of their role in autosomal dominant polycystic kidney disease (ADPKD) and an increasing range of so called ciliopathies.^13^

Primary cilia are now known to function as a hub for various signalling pathways including mechanotransduction, as well as many pathways whose receptors are localized in the cilium such as Wnt and Hedgehog signalling.^14^, ^15^, ^16^ More recent studies have shown involvement in growth factor signalling, differentiation, and inflammation.^17^, ^18^ These pathways are important for development as well as healthy cell and tissue function.^19^

Although cilia have been identified in tendon and shown to elongate in response to stress deprivation, the mechanism responsible for elongation, and the further consequences, are still unclear. Furthermore, no previous studies have investigated primary cilia expression or response to stress deprivation in the different regions of the tendon. Therefore, the aim of this study was to investigate how stress deprivation affects primary cilia in the fascicular and inter‐fascicular tendon matrix. We then evaluated whether changes in cilia are mediated by differential changes in the mechanical degradation in the two regions. Finally, we examined whether any localized changes in the cilia structure and tissue mechanics could be prevented by the application of static loading.

## METHODS

Tendon fascicles were obtained from the tails of 200 g (∼10–12 weeks old) female Sprague–Dawley rats, obtained from rats killed for unrelated purposes. Rats were euthanized by neck dislocation for extraction of dorsal root ganglia. Animal procedures were approved by the Animal Care Committee of Queen Mary, University of London, and the UK Home Office, and were in accordance with the UK Animals (Scientific Procedures) Act of 1986, and with the EU Directive 2010/63/EU for the protection of animals used for scientific purposes. Fascicles were obtained by removing the skin of the proximal end of the tail, after which the tendon was cut at each end and the fascicles pulled out taking care to ensure that the IFM surrounding each fascicle was maintained. Fascicles in the rat tail are easy to distinguish and are usually organized in groups of 2–3 fascicles within a sheath.

Fascicles were used either immediately (fresh group) or incubated in DMEM with the addition of 1.85 g sodium bicarbonate per every 500 ml, 10% foetal calf serum, 2% HEPES buffer, 1%L‐Glutamine, 1% non‐essential amino acids, 96 μg/ml penicillin, and 96μg/ml streptomycin. Unless stated otherwise, fascicles were incubated for 7 days, either maintained unconstrained in media in individual wells (stress deprived group) or clamped at the fascicle ends and strained to 4% strain and held at this value in a custom built chamber (4% static strain group).^20^ A figure showing a fascicle clamped in a chamber has been included in Supplementary Figure S1.

### Cilia Imaging

A total of 18 fascicles from three separate rats were prepared for each of the three test groups. After preparation, fascicles were fixed immediately in 100% methanol for 2 h at room temperature before washing in phosphate buffered saline (PBS) with 0.1% bovine serum albumin (BSA).

All antibodies were diluted in PBS with 0.1% BSA. Fascicles were incubated overnight at 4°C with rabbit anti‐arl13b (1:100, Abcam, Cambridge, UK) to stain the cilia membrane, after which they were washed and then incubated for 1 h at room temperature with a 488 nm Alexa secondary antibody (1:1000, Molecular Probes, Invitrogen, Eugene, OR). Finally samples were washed prior to incubation with 4,6‐diamidino‐2‐phenylindole for 5 min at room temperature (1:5000, Molecular Probes, Invitrogen). Fascicles were mounted under glass and confocal stack images were taken using a Leica Laser Scanning Confocal TCS SP2 with a 63× oil immersion lens. Stacks were taken with slices 250 nm apart in the z direction through half the thickness of the fascicle at a pixel resolution of 116 × 116 nm. Cilia lengths were measured in maximal projection using ImageJ. Since maximal projections underestimate cilia length due to their potential orientation out of the confocal plane the number of z slices in which the cilia was observed was counted and this was used to reconstruct the full cilia length using Equation (1). This equation assumes that the cilium length may be calculated as the hypotenuse of a triangle where the other two sides are the projected length in the xy plane and the vertical z distance between the base and the tip of the cilium. The latter is calculated based on the number of confocal xy sections in which the cilium appears multiplied by the z step size. In addition, the relatively poor z‐resolution means that it is necessary to adjust this z distance by the limit of z‐resolution. A schematic demonstrating this equation has been included in the Supplementary Figure S2.

(1) $$Corrected\;Length=\sqrt[{2}]{{\left((n-1)\Delta z-\delta z-t\right)}^{2}+L^{2}}$$

Equation (1): Reconstructing Cilia Length From a 3D Image Stack. Where *L* is the length of the cilium in the maximal projection image; *n* is the number of sections in which the cilium was observed; Δ*z* is the z step size (i.e., the distance between z sections); *t* is the thickness of the cilium which has been estimated at 0.2 µm^21^; and δ*z* is the limit of the z‐resolution of the objective lens. The limit of resolution is based on the full width half maximum (FWHM) distance of the point spread function in the z direction and is approximately 0.5 µm for the ×63/0.95 NA objective used in these studies.

### Time Course for Cilia Length Changes

The time frame over which cilia length changes occurred was also investigated. Twenty‐four fascicles were dissected from a single tail. Six fascicles were prepared for imaging immediately with further groups of six fascicles first subjected to either, 6, 16, or 24 h stress deprivation prior to imaging. Each group was fixed, stained and imaged following the protocol previously described, and the data combined with that collected after 7 days stress deprivation from the previous experiment.

### Fascicle and IFM Mechanics

Fascicle mechanics in the fresh, stress deprivation, and static strain groups were tested using a mechanical testing machine (ElectoPuls 1000; Instron, Canton, MA). Samples were prepared under each condition as previously described (*n* = 8 fascicles per group). Fascicle diameter was measured using a laser micrometer (LSM 501; Mitutoyo, Kawasaki, Kanagawa, Japan) with the lowest diameter across the test length recorded. Fascicles were secured in pneumatic grips at a gripping pressure of 4 bar and a grip separation distance of 20 mm. A tare load of 0.1 N was applied to each sample, and the sample length established for strain calculations. Fascicles were preconditioned by applying 10 cycles between 0% and 4% strain at 1 Hz, followed by a strain to failure test at an extension rate of 1 mm/s. Quasi static failure properties were calculated from the extension to failure test and hysteresis and cyclic stress relaxation were calculated from the preconditioning cycles. Hysteresis was calculated for each cycle as the difference in area beneath the loading and unloading curve, while stress relaxation was calculated as the percentage reduction in peak stress from the first to the tenth cycle.

IFM mechanics were investigated using our previously described shear model.^22^ Briefly, fascicles were dissected in attached pairs, and opposing ends of the two fascicles were cut at a separation of 20 mm, such that the only mechanism of transmitting force from one end of the sample to the other was through IFM shear. The opposing fascicle ends were then secured in the pneumatic grips and a 0.05 N tare load was applied to the samples. Samples were then strained to failure at 1 mm/s. Fresh and stress deprived groups were both tested (*n* = 6 fascicles per group). However, it was not possible to test 4% statically strained IFM samples since clamping and holding the fascicle pairs under static strain during sample preparation caused the IFM to tear and the fascicles to separate. Therefore only the fresh and stress deprived test groups are reported.

### Data Analysis

All data was assessed for normality with the Shapiro–Wilk test and then tested for significance with one tailed unpaired Student's *t*‐tests. A full table of tests and conditions is included in Supplementary Figure S3.

## RESULTS

### Cilia Length and Orientation

**Figure 2:**
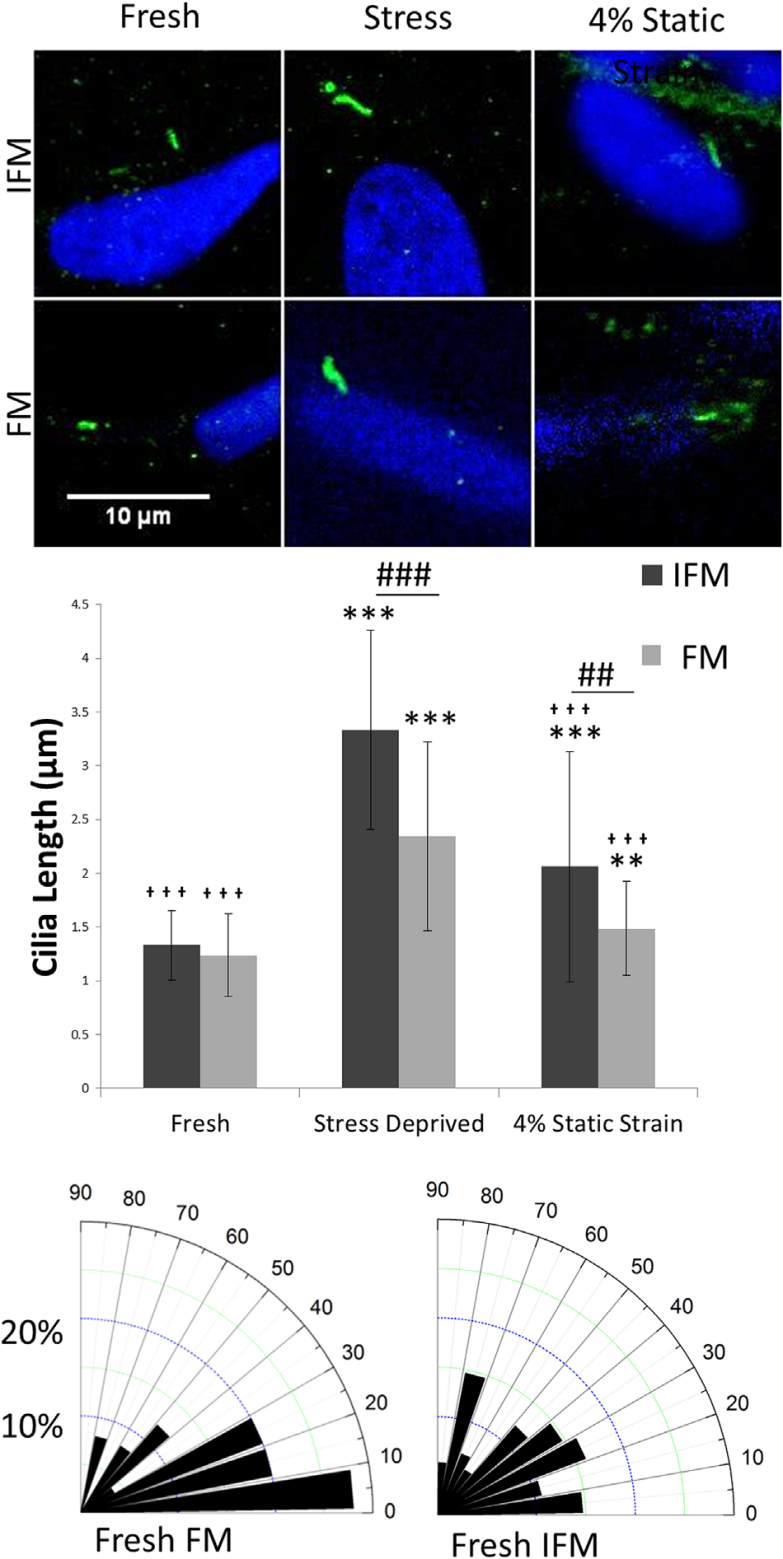
Under stress deprivation cilia length increases more in the inter‐fascicular matrix than in the fascicular matrix. Under static strain the cilia length is partially conserved. (a) Shows representative cilia for each condition. (b) Shows mean cilia length in each condition. Cilia measurements from three rat tails were combined. *N* = 100 ± 20 cilia measured per condition. Asterisks (*) show significance from the same region in fresh tissue. Crosses (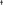) show significance from the same region in stress deprived tissue. Hashes (#) show significance between the two tendon regions for a given condition. ^*,^ 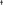 ^,#^ *p* < 0.05, ^**,^ 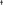 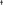 ^,##^ *p* < 0.01, ^***,^ 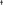 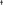 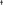 ^,###^ *p* < 0.001. Error bars show ±1 standard deviation. Statistical analysis performed using 1‐tailed, Student's *t*‐test. (c) Shows frequency distribution rose histograms of cilia angle with respect to the long axis of the tendon in fresh tissue.

Representative images of FM and IFM cells in all three test conditions are shown in Figure 2a, from which mean cilia lengths were calculated (Fig. 2b). Confirming previous work, cilia in stress deprived fascicles were shown to significantly increase in length.^23^ However, data additionally showed that maintaining fascicles under static strain resulted in a significant reduction in cilia length relative to stress deprived tissue. Considering regional differences in cilia length, there was no significant difference in cilia length between IFM and FM cells in fresh samples. However, in the stress deprived tissue, IFM cell cilia almost trebled in length and were significantly longer than the cilia of fascicular cells. Maintaining the samples under 4% static strain for 7 days lead to significantly reduced length increase compared with the stress deprived tissue for both the IFM and FM cells. However, there was still a significant increase in cilia length in both groups relative to the fresh tissue.

In fresh FM cells, cilia were predominantly orientated in the direction of normal axial tendon loading, which has been previously observed.^24^ However, there was no predominant orientation observed in IFM cells (Fig. 2c). While there did appear to be some loss of alignment of FM cilia with stress deprivation, this was not significant (data not shown).

**Figure 3:**
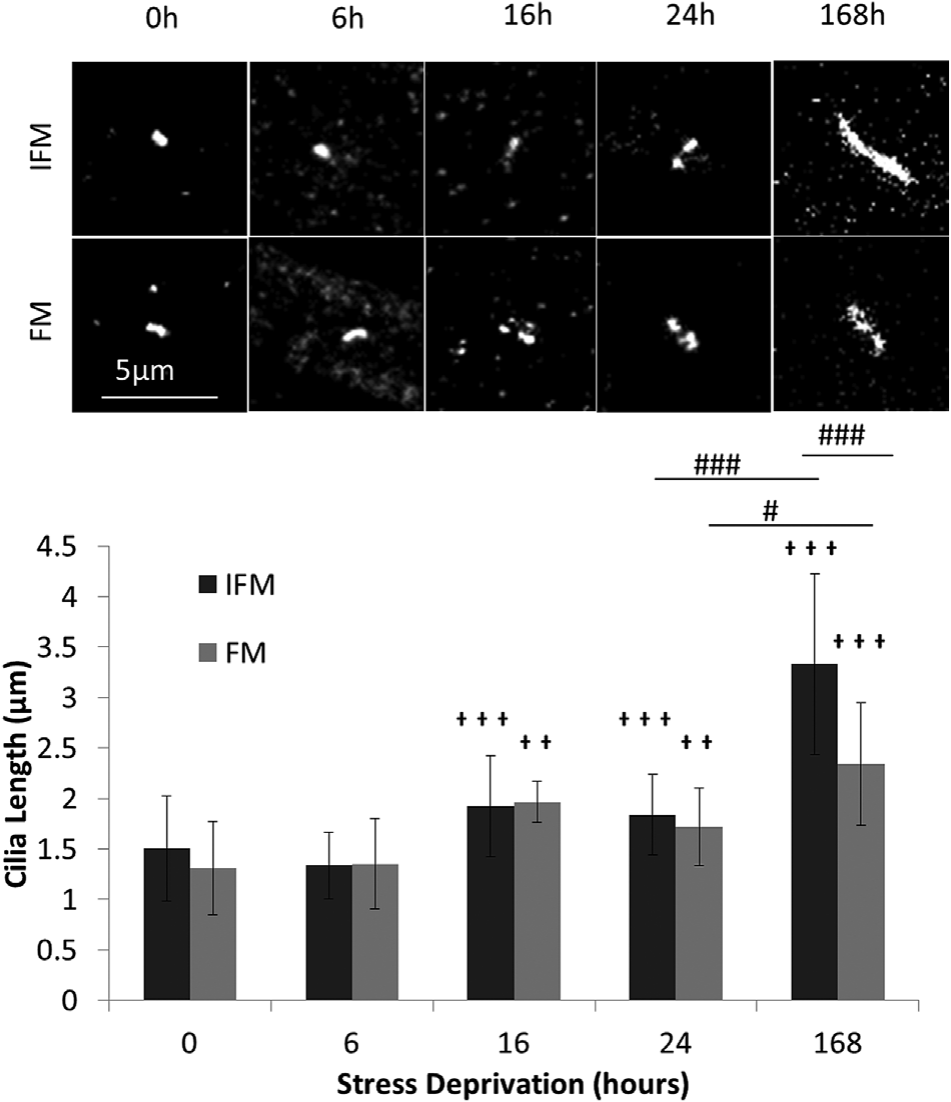
Stress deprivation time course shows that initially cilia length increases at the same rate in FM and IFM cells but after 1 week IFM cell cilia are considerably longer than FM cell cilia. (a) Shows representative cilia in the IFM & FM for each time point. (b) Shows mean cilia length in fascicular and IFM cells after 0, 6, 16, 24, and 168 h of stress deprivation. *N* > 40 cilia measured per condition. Crosses (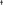) show significance from the same region in 0 h tissue. Hashes (#) show significance between the two tendon regions for a given condition. ^*,^ 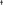 ^,#^ *p* < 0.05, ^**,^ 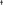 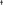 ^,##^ *p* < 0.01, ^***,^ 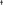 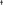 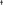 ^,###^ *p* < 0.001. Error bars show ±1 standard deviation.

A time course was performed in order to gain some insights into potential lengthening mechanisms for the cilia of both regions (Fig. 3). No increase in cilia length in either IFM or FM cells was observed after 6 h of stress deprivation. However, by 16 h, stress deprivation had led to a significant increase in cilia length in both the fascicular and IFM cells. Data indicates that the increase in FM cell cilia length occurred gradually over the time course, while the majority of the IFM cell cilia length increase occurred after 24 h.

### Fascicle and IFM Mechanics

**Figure 4:**
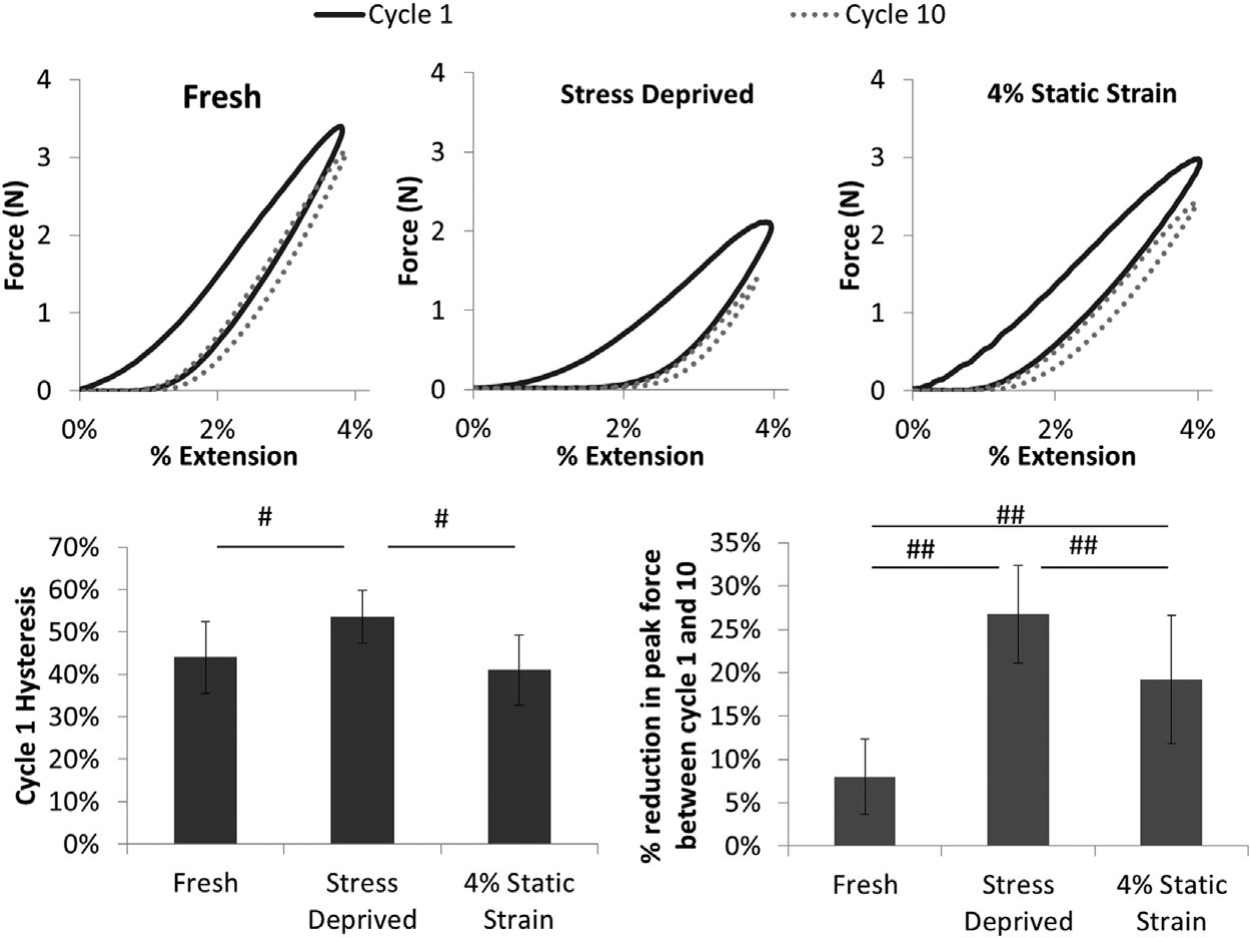
Under stress deprivation there is significant increase in viscoelastic behavior. (a–c) Show representative 4% cyclic preconditioning loops for fresh, stress deprived, and 4% static strained fascicles, respectively. Each figure shows preconditioning cycles 1, 2, and 10. (d) Shows the mean hysteresis in the first preconditioning cycle. (e) Shows the mean percentage reduction in peak force over the 10 preconditioning cycles. *N* = 8 fascicles per group. ^#^ *p* < 0.05, ^##^ *p* < 0.01, ^###^ *p* < 0.001. Error bars show ±1 standard deviation.

Figure 4a shows representative preconditioning loops for fresh, stress deprived, and 4% static strain fascicles, from which hysteresis and stress relaxation were calculated. Hysteresis in cycle 1 is significantly higher in the stress deprived fascicles than in the other two conditions (Fig. 4b). Furthermore, stress relaxation over the 10 cycles was significantly higher in the stress deprived fascicles than in either of the other two conditions (Fig. 4c). However, a significant increase in stress relaxation was also seen in the 4% static strain condition relative to the fresh control.

**Figure 5:**
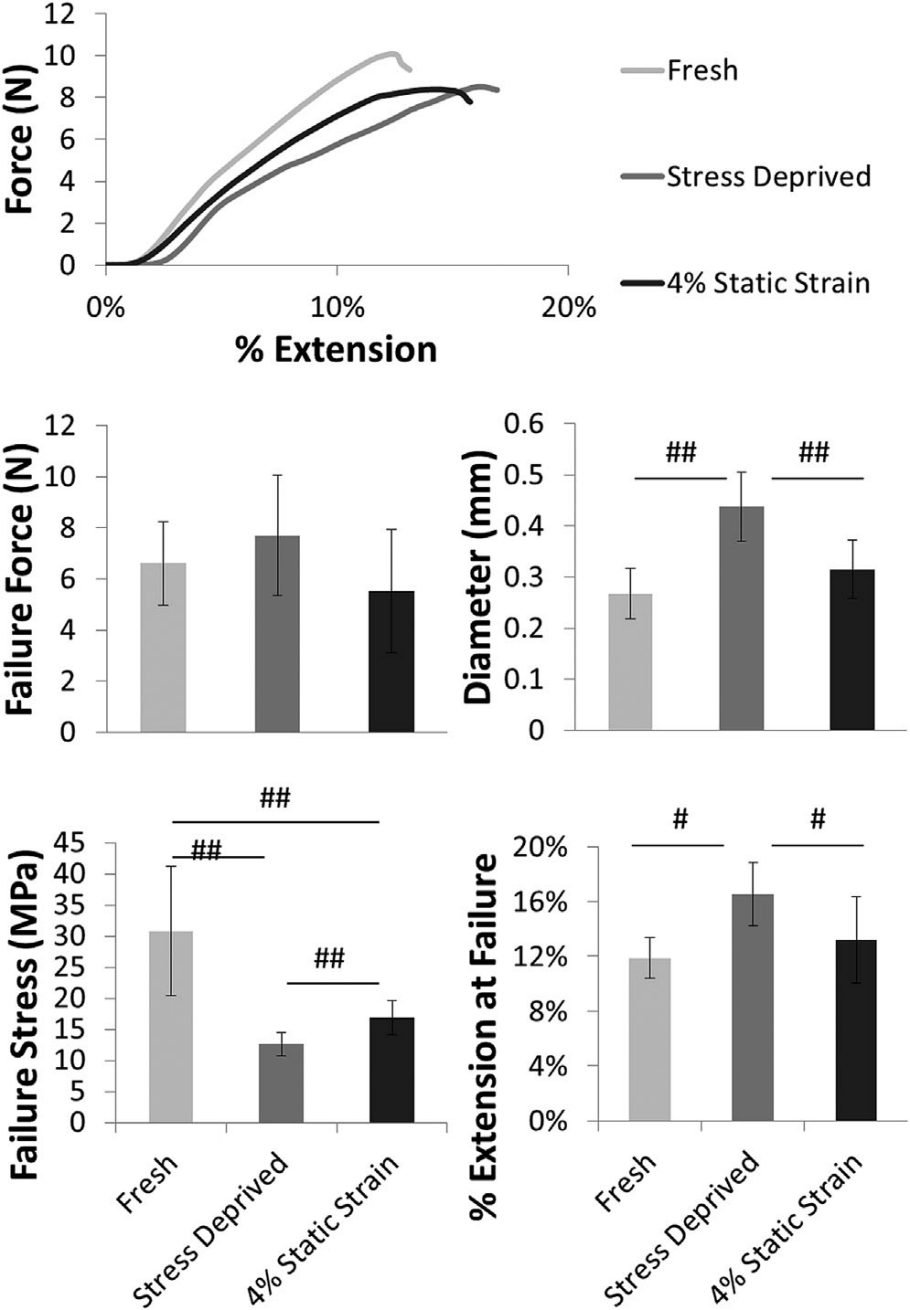
Stress deprivation does not cause significant loss of quasi static mechanical properties in fascicles. (a) Shows representative failure curves for each condition, from which mean fascicle failure force, diameter, failure stress, and failure strain are determined. Failure force is not significantly different for any of the groups (b); however, failure stress is significantly lower for the stress deprived and statically strained fascicles, as a result of the significant increase in average fascicle diameter. Failure extension was significantly higher in the stress deprived samples. ^#^ *p* < 0.05, ^##^ *p* < 0.01, ^###^ *p* < 0.001. Error bars show ±1 standard deviation.

Quasi‐static tests to failure of fascicles indicated little change in failure properties between test groups (Fig. 5). While the failure stress was significantly reduced in stress deprived fascicles (Fig. 5d), it is evident from the consistent failure force across groups (Fig. 5b), that this was due to the increased fascicle diameter (Fig. 5c) and not a change in fascicle mechanics. However, failure strain was significantly higher in stress deprived fascicles compared with the other two conditions (Fig. 5e).

**Figure 6:**
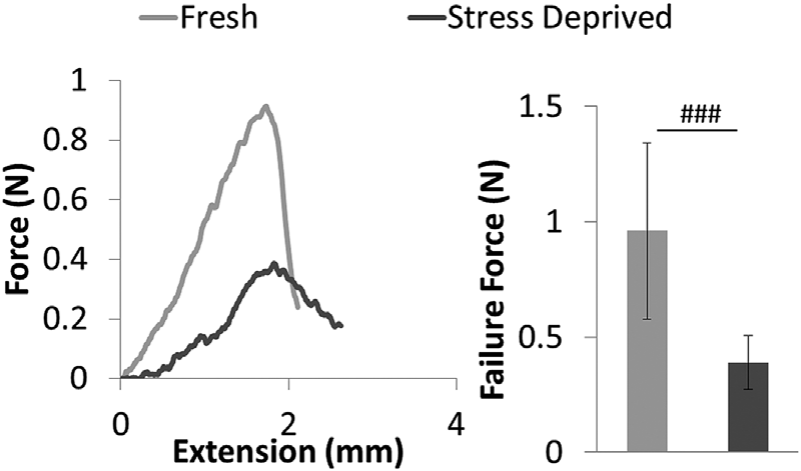
The inter‐fascicular matrix shows a significant reduction in failure strength after stress deprivation. This figure shows representative force‐extension curves to failure for the IFM in stress deprived and fresh samples (a), alongside mean IFM failure force data (b). *N* = 6 fascicle pairs per group. ^###^ *p* < 0.001. Error bars show ±1 standard deviation.

Mechanical changes to the IFM with stress deprivation were also investigated using the IFM shear model described above. Mechanical integrity of the IFM was reduced to such an extent that preconditioning was not possible for the stress deprived samples. Therefore, all samples were only subjected to a quasi‐static failure test, with typical curves shown in Figure 6a. After stress deprivation, the IFM fails at a significantly lower force, able to withstand only 50% of the failure force of fresh samples (Fig. 6b). However, the effect on the IFM of static loading is not known.

## DISCUSSION

It has been understood for some time that the tenocytes within functionally different tendons have different levels of metabolic activity.^25^ However, recent work from our group has shown that within a single tendon there are also metabolic and morphological differences between the cells in the fascicular matrix (FM) and the inter‐fasicular matrix (IFM).^22^, ^26^, ^27^ These differences include increased metabolic activity in IFM cells, which have also been previously observed to be rounder and more numerous for a given area, a finding that was also observed during this study (Fig. S4).^2^, ^8^ It is likely the IFM cell population is heterogeneous, but may contain a sub‐population of more metabolically active Tendon Derived Stem Cells (TDSCs).^28^

This study is the first to show that the primary cilia response to stress deprivation is different in the inter‐fascicular and fascicular matrix, and that this reflects differences in the biomechanical degradation of these regions. In particular, we show that stress deprivation causes greater elongation of the primary cilia in the inter‐fascicular matrix associated with a greater loss of biomechanical integrity in this region.

In fresh tendon, primary cilia length was the same in both the inter‐fascicular matrix (IFM) and the fascicular matrix (FM) with most cilia between 1 and 2 μm.We did not attempt to quantify cilia prevalence, as it is known that short cilia (<1 μm in length) are difficult to identify in intact tissue, such that population estimates of cilia length tendon to be biased towards longer cilia, and any attempt to quantify cilia prevalence is likely to produce an underestimate of the true prevalence, particularly for samples with shorter cilia. In the fascicular region, cilia were oriented in the direction of the long axis of the tendon, which is also the axis of applied mechanical load. By contrast, cilia in the inter‐fascicular region were distributed with no specific orientation. These differences in cilia orientation may reflect the differences in the local strain environment of the cells. Alternatively, cilia orientation may be determined by the structural organization of the collagen matrix, as seen in previous studies, which have demonstrated that cilia alignment is associated with nanoscale topographical organization of the substrate.^29^ Indeed, in agreement with previous studies, under stress deprivation the highly aligned cilia in fascicular cells appeared to become more randomly orientated,^30^ potentially reflecting the loss of collagen organization typically reported with stress deprivation.^31^

While it has been previously shown that tenocyte cilia lengthen under stress deprivation in the FM,^23^ we have shown for the first time that after 7 days of stress deprivation, cilia in the IFM show a significantly greater elongation; 151% compared to only 90% in the FM. The mechanism responsible for this cilia elongation is unclear, although there are many regulators of cilia length including actin tension, the availability of soluble tubulin and direct physical stimuli.^32^, ^33^ Previous studies have reported that increased mechanical loading results in cilia shortening in chondrocytes via a mechanism involving the tubulin deacetylase, histone deacetylase 6 (HDAC6)^34^ as also occurs in response to heat shock.^35^ It is therefore possible that stress deprivation causes a reciprocal relationship, inhibiting the effect of HDAC6 thereby enabling cilia elongation.

Time course studies demonstrate that lengthening is not detectable until 16 h of stress deprivation, with the majority of elongation occurring beyond 24 h. This suggests that lengthening is not caused solely by the removal of mechanical load, since it has been previously demonstrated that cilia can disassemble or elongate within 3–6 h of the removal of strain or other physicochemical stimuli.^18^, ^36^, ^37^ Therefore, we investigated whether cilia elongation was mediated by the upregulation of inflammatory cytokines such as IL‐1β seen during tendon stress deprivation,^38^ which have been shown to cause rapid primary cilia elongation in chondrocytes.^18^ However, in the current study no significant cilia elongation was observed after 6 h of treatment with IL‐1β in either the FM or IFM (Fig. S5). Therefore it seems unlikely that IL‐1β is directly responsible for the stress deprivation induced increase in cilia length. However, it may still be the case that other cytokines upregulated in stress deprivation may be responsible.

With our time course evidence suggesting that the length changes were not caused by direct changes in strain and, having shown stress deprivation does not induce IL‐1 β, we next examined whether cilia elongation was associated with the biomechanical and structural degradation of the matrix, by quantifying the changes in mechanics in both regions during stress relaxation. The lower shear strength of the IFM in the rat tail compared to that seen in other more functionally loaded tendons,^27^ does limit the applicability of the rat tail model for investigating IFM and FM mechanical changes. However, its extensive use over the last 50 years, and the importance of being able to prepare samples for both cilia imaging and mechanical characterization without dissection damage to either the IFM or FM makes it the most suitable model for this particular investigation.^39^, ^40^ We found that the biomechanical properties of the IFM and the FM change differently under stress deprivation. In the FM, there was no loss in fascicular failure force although significant swelling of the fascicle occurred, which caused a reduction in failure stress due to increased cross sectional area. Swelling was probably also responsible for the increased failure strain and viscoelasticity of the fascicles, as more extensive fluid movement will increase the capacity for energy dissipation in the matrix during loading.^41^ By contrast, much larger biomechanical changes were observed in the IFM where the failure force of the IFM in shear reduced significantly, by approximately 50%, after stress deprivation.

A number of previous studies, while not directly associating stress deprivation with IFM degradation, do strongly support such a finding. Stress deprivation has been shown to affect the transverse mechanical properties of tendon more substantially than the longitudinal properties^42^ and also to affect whole tendon sections more than isolated fascicles,^43^ pointing towards the greatest stress deprivation changes occurring in the matrix between fascicles. Further, stress deprivation has been shown to more adversely affected non‐collagenous that collagenous matrix components,^44^ which would also lead to a greater degeneration of the IFM, which is known to be proteoglycan‐rich.^26^

Taken together, there is a clear correlation between biomechanical matrix degradation and the extent of primary cilia elongation. Thus, our data indicates that mechanical degradation of the ECM may directly contribute to the cilia elongation associated with stress deprivation. However, the precise pathways associating these parameters remain unclear. The matrix degradation occurring with stress deprivation is likely cell mediated. However, studies have reported some loss of mechanical properties even in acellular tendon samples subjected to stress deprivation.^45^ While not directly influencing cilia length IL‐1β may still play a role in directly initiating tendon mechanical degradation. Treatment using IL‐1β antagonists slightly reduces the biomechanical degradation of whole tendon samples during stress deprivation.^46^ However, IL‐1β antagonists did not significantly reduce degradation of individual fascicles, suggesting that IL‐1β mediated degradation is primarily localized to the IFM. This correlates with studies showing that IL‐6, MMP‐3, and MMP‐13 are all upregulated in response to tendon overload, but specifically in IFM cells, suggesting more pronounced metabolic changes are localized to the IFM region.^8^

While the elongation of the cilia seems to be induced, at least in part, by matrix degradation, the consequences of this elongation are unclear. In other cell types, cilia elongation is associated with increased mechanotransduction,^47^ reduced hedgehog signalling,^48^ reduced Wnt signalling,^49^ and increased inflammatory signalling.^16^, ^18^, ^50^ As such, changes in cilia length caused by matrix degradation may subsequently influence cilia signalling pathways and further modulate the biomechanical degradation.

To further investigate the relationships between stress deprivation, matrix degeneration, and cilia lengthening, we attempted to prevent both the cilia elongation and biomechanical degradation by the application of a gross static strain over the same 7‐day period. Unfortunately, it was not possible to test the mechanical properties of the statically strained IFM, since the process of clamping and statically straining two attached fascicles caused them to separate over the 7‐day culture period.

However, maintaining the fascicles at 4% static strain was shown to minimize the biomechanical changes seen in the fascicles. Furthermore, static strain conditions significantly reduced the cilia elongation in both the FM and the IFM. This agrees with previous work that shows that tendon requires loading in order to properly function and that the loss of this stress causes tendon degradation.^3^, ^51^

In summary, we show for the first time that tendon stress deprivation explicitly leads to greater mechanical degradation in the IFM compared with the FM and that this correlates with a greater increase in primary cilia length. We suggest that stress deprivation drives matrix catabolism, possibly partly through the up‐regulation of inflammatory signalling, and that the resulting biomechanical and structural degradation drives the primary cilia reorientation and elongation. The greater loss of biomechanical integrity with stress deprivation in the IFM is consistent with previous reports that the IFM turns over more rapidly than the FM in tendon^2^; hence, any cellular remodelling is likely to occur faster in the IFM. Furthermore, increased expression of MMPs and inflammatory cytokines are seen localized to this region with loading, again indicating a more active cell population.^8^

The reported differential changes in cilia structure following stress deprivation are likely to produce differences in cilia signalling which may further modulate the differential degradation between tendon regions. Such zonal changes in cilia structure and function may therefore have important consequences in the aetiology of tendinopathy. Furthermore, in other connective tissues such as ligaments, intervertebral disks, articular cartilage, or even blood vessels, similar biomechanical degradation induced by physicochemical or pathological stimuli may lead to similar alterations in primary cilia structure, thereby modulating cell behavior.

## AUTHORS’ CONTRIBUTIONS

Research study design, data interpretation, critical revision of paper, approval of final and submitted version—MK and HS. Research study design, data acquisition and analysis, data interpretation, drafting the paper, approval of final and submitted version—DR.

## Supporting information

Additional supporting information may be found in the online version of this article at the publisher's web‐site.

**Table S1**. Cell straining chamber with clamped fascicle.

**Table S2**. Schematic of cilia measurement in projection and corrected length.

**Table S3**. Table detailing all experiments performed and their condition and outputs.

**Table S4**. Nuclei of IFM cells are significantly more rounded than nuclei of FM cells.

**Table S5**. Treatment with IL1 does not induce cilia lengthening in ex vivo tendon fascicle or IFM.

## References

[1] Buchanan CI , Marsh RL . 2002 Effects of exercise on the biomechanical, biochemical and structural properties of tendons. Comp Biochem Physiol 133:1101–1107. 10.1016/s1095-6433(02)00139-3 12485694

[2] Thorpe CT , Chaudhry S , Lei II , et al. 2015 Tendon overload results in alterations in cell shape and increased markers of inflammation and matrix degradation. Scand J Med Sci Sports 25:e381–e391. 25639911 10.1111/sms.12333

[3] Majima T , Yasuda K , Fujii T , et al. 1996 Biomechanical effects of stress shielding of the rabbit patellar tendon depend on the degree of stress reduction. J Orthop Res 14:377–383. 8676249 10.1002/jor.1100140306

[4] Thornton GM , Shao X , Chung M , et al. 2010 Changes in mechanical loading lead to tendonspecific alterations in MMP and TIMP expression: influence of stress deprivation and intermittent cyclic hydrostatic compression on rat supraspinatus and Achilles tendons. Br J Sports Med 44:698–703. 18801769 10.1136/bjsm.2008.050575

[5] Yamamoto N , Ohno K , Hayashi K , et al. 1993 Effects of stress shielding on the mechanical properties of rabbit patellar tendon. J Biomech Eng 115:23. 8445894 10.1115/1.2895466

[6] Sun Y‐L , Thoreson AR , Cha SS , et al. 2010 Temporal response of canine flexor tendon to limb suspension. J Appl Physiol 109:1762–1768. 20947711 10.1152/japplphysiol.00051.2010 PMC3006401

[7] Lavagnino M , Arnoczky SP , Egerbacher M , et al. 2006 Isolated fibrillar damage in tendons stimulates local collagenase mRNA expression and protein synthesis. J Biomech 39:2355–2362. 16256123 10.1016/j.jbiomech.2005.08.008

[8] Spiesz EM , Thorpe CT , Chaudhry S , et al. 2015 Tendon extracellular matrix damage, degradation and inflammation in response to in‐vitro overload exercise. J Orthop Res 33:889–897. 25721513 10.1002/jor.22879 PMC4855636

[9] Sorokin SP . 1968 Reconstructions of centriole formation and ciliogenesis in mammalian lungs. J Cell Sci 3:207–230. 5661997 10.1242/jcs.3.2.207

[10] Sorokin S . 1962 centrioles rudimentary and smooth and the formation of cilia muscle by fibroblasts. J Cell Biol 15:363–377. 13978319 10.1083/jcb.15.2.363 PMC2106144

[11] Kowalevsky A . 1867 Entwickelungsgeschichte des Amphioxus lanceolatus. Mem Acad Sci St Petersb 11:1–17.

[12] Beales P , Jackson PK . 2012 Cilia—the prodigal organelle. Cilia 1:1. 23351984 10.1186/2046-2530-1-1 PMC3541540

[13] Kathem SH , Mohieldin AM , Nauli SM . 2013 The roles of primary cilia in polycystic kidney disease. AIMS Mol Sci 1:27–46. 10.3934/molsci.2013.1.27 PMC4296740 25599087

[14] Nauli SM , Alenghat FJ , Luo Y , et al. 2003 Polycystins 1 and 2 mediate mechanosensation in the primary cilium of kidney cells. Nat Genet 33:129–137. 12514735 10.1038/ng1076

[15] Rohatgi R , Milenkovic L , Scott MP . 2007 Patched1 regulates hedgehog signaling at the primary cilium. Science 317:372–376. 17641202 10.1126/science.1139740

[16] Wann AKT , Zuo N , Haycraft CJ , et al. 2012 Primary cilia mediate mechanotransduction through control of ATP‐induced Ca2+ signaling in compressed chondrocytes. FASEB J 26:1663–1671. 22223751 10.1096/fj.11-193649 PMC3316893

[17] Schneider L , Clement CA , Teilmann SC , et al. 2005 PDGFRalphaalpha signaling is regulated through the primary cilium in fibroblasts. Curr Biol 15:1861–1866. 16243034 10.1016/j.cub.2005.09.012

[18] Wann AKT , Knight MM . 2012 Primary cilia elongation in response to interleukin‐1 mediates the inflammatory response. Cell Mol Life Sci 69:2967–2977. 22481441 10.1007/s00018-012-0980-y PMC3417094

[19] May‐Simera HL , Kelley MW . 2012 Cilia, Wnt signaling, and the cytoskeleton. Cilia 1:7. 23351924 10.1186/2046-2530-1-7 PMC3555707

[20] Screen HRC , Shelton JC , Bader DL , et al. 2005 Cyclic tensile strain upregulates collagen synthesis in isolated tendon fascicles. Biochem Biophys Res Commun 336:424–429. 16137647 10.1016/j.bbrc.2005.08.102

[21] Andrews PM . 1974 A scanning electron microscopic study of the nephron. Am J Anat 140:81–115. 4132935 10.1002/aja.1001400107

[22] Thorpe CT , Godinho MSC , Riley GP , et al. 2015 The interfascicular matrix enables fascicle sliding and recovery in tendon, and behaves more elastically in energy storing tendons. J Mech Behav Biomed Mater 52:85–94. 25958330 10.1016/j.jmbbm.2015.04.009 PMC4655227

[23] Gardner K , Arnoczky SP , Lavagnino M . 2011 Effect of in vitro stress‐deprivation and cyclic loading on the length of tendon cell cilia in situ. J Orthop Res 29:582–587. 20957738 10.1002/jor.21271

[24] Donnelly E , Ascenzi M‐G , Farnum C . 2010 Primary cilia are highly oriented with respect to collagen direction and long axis of extensor tendon. J Orthop Res 28:77–82. 19603516 10.1002/jor.20946 PMC2847399

[25] Birch HL , Worboys S , Eissa S , et al. 2008 Matrix metabolism rate differs in functionally distinct tendons. Matrix Biol 27:182–189. 18032005 10.1016/j.matbio.2007.10.004

[26] Thorpe CT , Birch HL , Clegg PD , et al. 2013 The role of the non‐collagenous matrix in tendon function. Int J Exp Pathol 94:248–259. 23718692 10.1111/iep.12027 PMC3721456

[27] Thorpe CT , Udeze CP , Birch HL , et al. 2012 Specialization of tendon mechanical properties results from interfascicular differences. J R Soc Interface 9:3108–3117. 22764132 10.1098/rsif.2012.0362 PMC3479922

[28] Lui PPY , Chan KM . 2011 Tendon‐derived stem cells (TDSCs): from basic science to potential roles in tendon pathology and tissue engineering applications. Stem Cell Rev 7:883–897. 21611803 10.1007/s12015-011-9276-0

[29] Mcmurray R , Knight MM . 2012 Topographical regulation of primary cilia orientation and length in mesenchymal stem cells. Cilia 1:P34.

[30] Lavagnino M , Arnoczky SP , Gardner K . 2011 In situ deflection of tendon cell‐cilia in response to tensile loading: an in vitro study. J Orthop Res 29:925–930. 21259338 10.1002/jor.21337

[31] Hannafin JA , Arnoczky SP , Hoonjan A , et al. 1995 Effect of stress deprivation and cyclic tensile loading on the material and morphologic properties of canine flexor digitorum profundus tendon: anin vitro study. J Orthop Res 13:907–914. 8544028 10.1002/jor.1100130615

[32] Miyoshi K , Kasahara K , Miyazaki I , et al. 2011 Factors that influence primary cilium length. Acta Med Okayama 65:279–285. 22037264 10.18926/AMO/47009

[33] Pitaval A , Tseng Q , Bornens M , et al. 2010 Cell shape and contractility regulate ciliogenesis in cell cycle‐arrested cells. J Cell Biol 191:303–312. 20956379 10.1083/jcb.201004003 PMC2958475

[34] Thompson CL , Chapple JP , Knight MM . 2014 Primary cilia disassembly down‐regulates mechanosensitive hedgehog signalling: a feedback mechanism controlling ADAMTS‐5 expression in chondrocytes. Osteoarthritis Cartilage 22:490–498. 24457103 10.1016/j.joca.2013.12.016 PMC3988976

[35] Prodromou NV , Thompson CL , Osborn DPS , et al. 2012 Heat shock induces rapid resorption of primary cilia. J Cell Sci 125:4297–4305. 22718348 10.1242/jcs.100545 PMC3516438

[36] McGlashan SR , Knight MM , Chowdhury TT , et al. 2010 Mechanical loading modulates chondrocyte primary cilia incidence and length. Cell Biol Int 34:441–446. 20100169 10.1042/CBI20090094

[37] Besschetnova TY , Kolpakova‐Hart E , Guan Y , et al. 2010 Identification of signaling pathways regulating primary cilium length and flow‐mediated adaptation. Curr Biol 20:182–187. 20096584 10.1016/j.cub.2009.11.072 PMC2990526

[38] Uchida H , Tohyama H , Nagashima K , et al. 2005 Stress deprivation simultaneously induces over‐expression of interleukin‐1beta, tumor necrosis factor‐alpha, and transforming growth factor‐beta in fibroblasts and mechanical deterioration of the tissue in the patellar tendon. J Biomech 38:791–798. 15713300 10.1016/j.jbiomech.2004.05.009

[39] Rigby BJ , Hirai N , Spikes JD , et al. 1959 The mechanical properties of rat tail tendon. J Gen Physiol 43:265–283. 19873525 10.1085/jgp.43.2.265 PMC2194989

[40] Bruneau A , Champagne N , Cousineau‐Pelletier P , et al. 2010 Preparation of rat tail tendons for biomechanical and mechanobiological studies. J Vis Exp 41:3–7. 10.3791/2176 PMC3156064 20729800

[41] Screen HRC , Seto J , Krauss S , et al. 2011 Extrafibrillar diffusion and intrafibrillar swelling at the nanoscale are associated with stress relaxation in the soft collagenous matrix tissue of tendons. Soft Matter 7:11243–11251.

[42] Yamamoto E , Hayashi K , Yamamoto N . 2000 Effects of stress shielding on the transverse mechanical properties of rabbit patellar tendons. J Biomech Eng 122:608–614. 11192382 10.1115/1.1319660

[43] Yamamoto E , Hayashi K , Yamamoto N . 1999 Mechanical properties of collagen fascicles from stress‐shielded patellar tendons in the rabbit. Clin Biomech (Bristol, Avon) 14:418–425. 10.1016/s0268-0033(99)00006-6 10521624

[44] Abreu EL , Leigh D , Derwin a. K . 2008 Effect of altered mechanical load conditions on the structure and function of cultured tendon fascicles. J Orthop Res 26:364–373. 17972327 10.1002/jor.20520

[45] Ohno K , Yasuda K , Yamamoto N , et al. 1993 Effects of complete stress‐shielding on the mechanical properties and histology of in situ frozen patellar tendon. J Orthop Res 11:592–602. 8340831 10.1002/jor.1100110414

[46] Miyatake S , Tohyama H , Kondo E , et al. 2008 Local administration of interleukin‐1 receptor antagonist inhibits deterioration of mechanical properties of the stress‐shielded patellar tendon. J Biomech 41:884–889. 18062978 10.1016/j.jbiomech.2007.10.018

[47] Spasic M , Jacobs C . 2015 Primary cilia length is critical to cellular mechanotransduction. Biophys J 108:561a.

[48] Thompson CL , Wiles A , Poole CA , et al. 2015 Lithium chloride modulates chondrocyte primary cilia and inhibits Hedgehog signaling. FASEB J 30:716–726. 26499268 10.1096/fj.15-274944

[49] McMurray RJ , Wann a KT , Thompson CL , et al. 2013 Surface topography regulates wnt signaling through control of primary cilia structure in mesenchymal stem cells. Sci Rep 3:25–28. 10.1038/srep03545 PMC3866595 24346024

[50] Wann A , Thompson C , Chapple J , et al. 2013 Interleukin‐1β sequesters hypoxia inducible factor 2α to the primary cilium. Cilia 2:1–14. 24330727 10.1186/2046-2530-2-17 PMC3886195

[51] Yamamoto E , Iwanaga W , Miyazaki H , et al. 2002 Effects of static stress on the mechanical properties of cultured collagen fascicles from the rabbit patellar tendon. J Biomech Eng 124:85. 11871609 10.1115/1.1427924

