# Supplementary figures and images for: Zonal variation in primary cilia elongation correlates with localized biomechanical degradation in stress deprived tendon
Source: J Orthop Res. 2016 Mar 23;34(12):2146–53. doi: 10.1002/jor.23229 (PMC5216897; doi:10.1002/jor.23229)

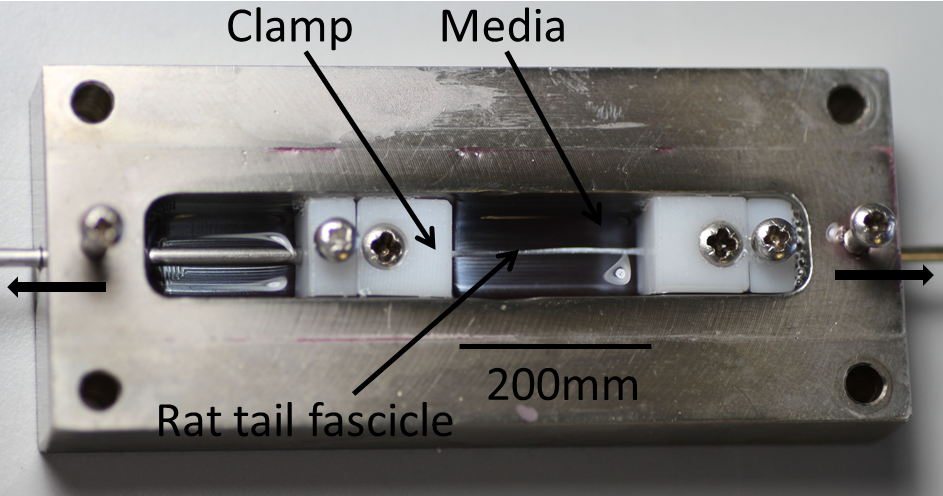

Supplement: Supplementary file 1 — Table S1. Cell straining chamber with clamped fascicle. [file JOR-34-2146-s001.tif]

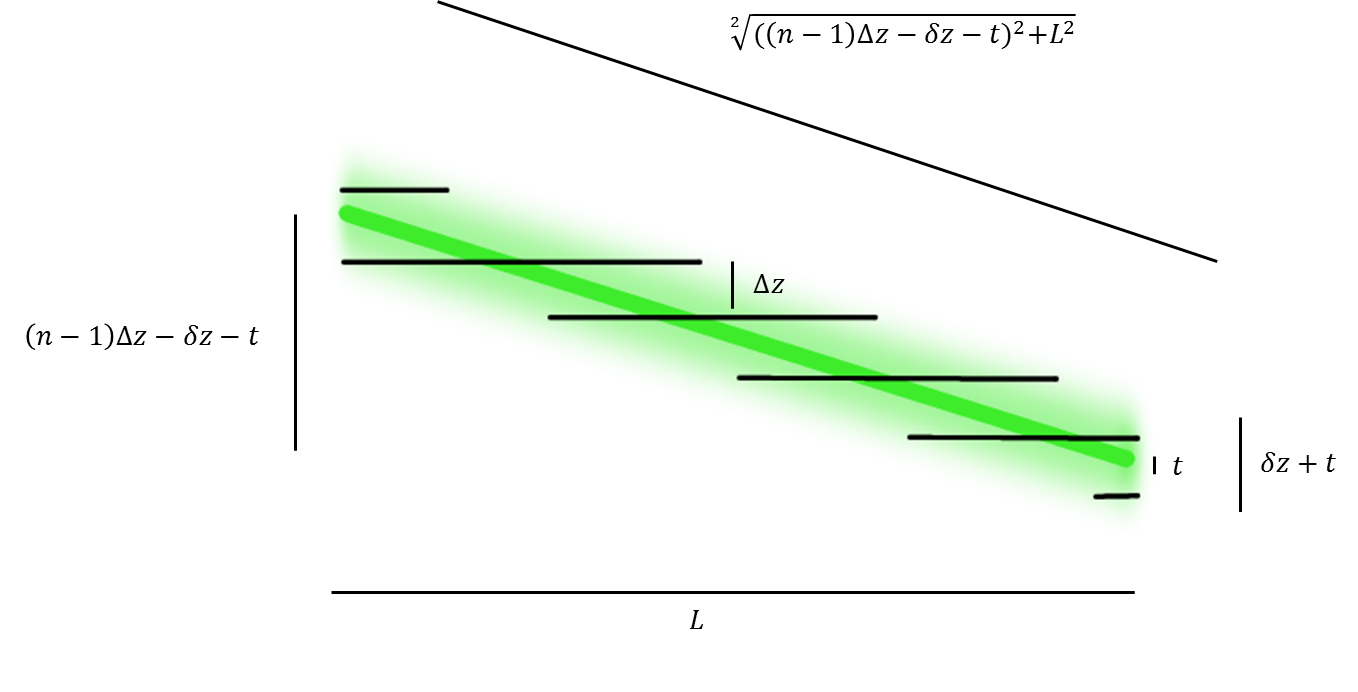

Supplement: Supplementary file 2 — Table S2. Schematic of cilia measurement in projection and corrected length. [file JOR-34-2146-s002.tif]

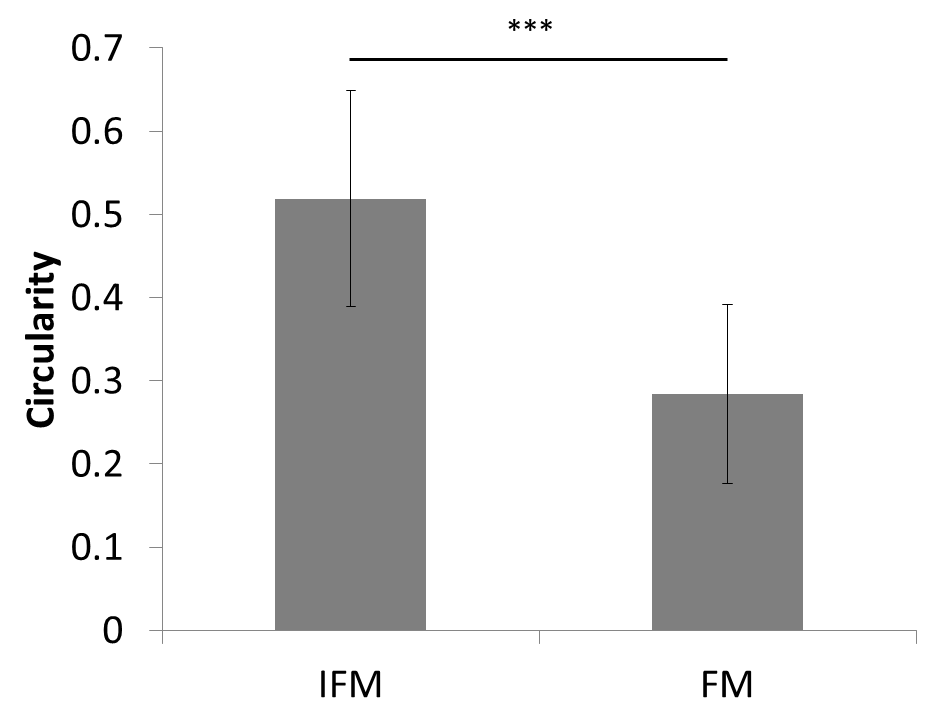

Supplement: Supplementary file 4 — Table S4. Nuclei of IFM cells are significantly more rounded than nuclei of FM cells. [file JOR-34-2146-s004.tif]

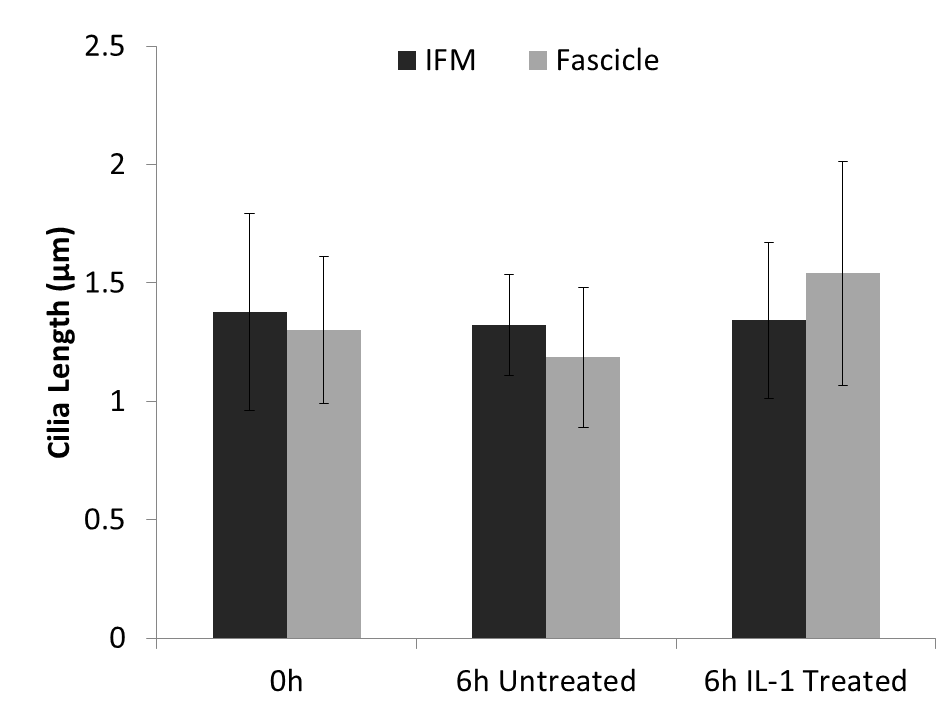

Supplement: Supplementary file 5 — Table S5. Treatment with IL1 does not induce cilia lengthening in ex vivo tendon fascicle or IFM. [file JOR-34-2146-s005.tif]
