# Supplementary material for: Zonal variation in primary cilia elongation correlates with localized biomechanical degradation in stress deprived tendon
Source: J Orthop Res. 2016 Mar 23;34(12):2146–53. doi: 10.1002/jor.23229 (PMC5216897; doi:10.1002/jor.23229)
Supplement: Supplementary file 3 — Table S3. Table detailing all experiments performed and their condition and outputs. [file JOR-34-2146-s003.docx]

| **Experiment** | **Conditions** | **Measurements** |
| --- | --- | --- |
| 7 Day Cilia Length Experiment  (Figure 2) | Fresh  Stress Deprived  4% Static Strain | Cilia Length  Cilia Orientation |
| Cilia Length Time Course  (Figure 3) | Fresh  Stress Deprived | Cilia Length |
| Fascicle Mechanics  (Figures 4 & 5) | Fresh  Stress Deprived  4% Static Strain | Diameter (fig. 4)  Failure Stress (fig. 4)  Failure Force (fig. 4)  Failure Strain (fig. 4)  Hysteresis across 10 cycles (fig. 5)  Stress relaxation over 10 cycles (fig. 5) |
| IFM Mechanics  (Figure 6) | Fresh  Stress Deprived | Failure Force |
